# Immune Checkpoint Blockade to Improve Tumor Infiltrating Lymphocytes for Adoptive Cell Therapy

**DOI:** 10.1371/journal.pone.0153053

**Published:** 2016-04-06

**Authors:** Krithika N. Kodumudi, Jessica Siegel, Amy M. Weber, Ellen Scott, Amod A. Sarnaik, Shari Pilon-Thomas

**Affiliations:** Department of Immunology, Moffitt Cancer Center, 12902 Magnolia Drive, SRB-3, Room 24314, Tampa, FL, 33612, United States of America; University of Colorado Denver, UNITED STATES

## Abstract

Tumor-infiltrating lymphocytes (TIL) has been associated with improved survival in cancer patients. Within the tumor microenvironment, regulatory cells and expression of co-inhibitory immune checkpoint molecules can lead to the inactivation of TIL. Hence, there is a need to develop strategies that disrupt these negative regulators to achieve robust anti-tumor immune responses. We evaluated the blockade of immune checkpoints and their effect on T cell infiltration and function. We examined the ability of TIL to induce tumor-specific immune responses *in vitro* and *in vivo*. TIL isolated from tumor bearing mice were tumor-specific and expressed co-inhibitory immune checkpoint molecules. Administration of monoclonal antibodies against immune checkpoints led to a significant delay in tumor growth. However, anti-PD-L1 antibody treated mice had a significant increase in T cell infiltration and IFN-γ production compared to other groups. Adoptive transfer of *in vitro* expanded TIL from tumors of anti-PD-L1 antibody treated mice led to a significant delay in tumor growth. Blockade of co-inhibitory immune checkpoints could be an effective strategy to improve TIL infiltration and function.

## Introduction

Infiltration of T cells into tumors has been correlated with improved survival in cancer patients. Although T cells are able to adequately infiltrate tumors, they are ineffective at eradicating patients’ tumors. Studies have shown that T cell infiltration in to tumor tissues are associated with reduction in the tumor burden and improved clinical prognosis [1,2]. Over the last decade, adoptive transfer of tumor infiltrating lymphocytes (TIL) has emerged as a promising approach to induce effective anti-tumor immunity and tumor regression in various cancers [3,4]. TIL therapy resulted in objective response rate of 40–50% in treated melanoma patients. [5,6]. Tumor-specific T cells may be inactivated *in vivo* by immunosuppressive factors in the local tumor microenvironment, such as T-regulatory and myeloid derived suppressor cells, or by signaling through of co-inhibitory molecules that modulate T cell activation. There are an increasing number of co-inhibitory signals in the tumor microenvironment that have been demonstrated to inhibit anti-tumor T cell responses. Activated T cells express multiple co-inhibitory receptors including lymphocyte activations gene 3 (LAG-3), B and T lymphocyte attenuator (BTLA), cytotoxic T lymphocyte antigen 4 (CTLA-4), and programmed death (PD-1) [7–11]. While these immune checkpoint receptors maintain T cell homeostasis, when expressed by tumor-specific T cells, they represent a significant barrier for the induction of effective anti-tumor immune responses. Blockade of these receptors has been shown to improve anti-tumor immune T cell responses. CTLA-4 (CD152) is a cell surface molecule that is expressed on activated T cells. Ipilimumab, approved by the FDA in 2011, targets the CTLA-4 receptor [12]. Lag-3 is a cell-surface molecule that is involved in T cell activation and function [13]. Studies have shown that LAG-3 is expressed on T regulatory cells (Tregs) and blockade of LAG-3 affects Treg function [14]. It has been shown that an in vitro suppression assay using CD4+CD25highLAG-3+ T cells showed that this subset of cells is endowed with potent suppressor activity and their frequency is enhanced in the PBMCs of patients with cancer and is expanded at tumor sites [15]. LAG3 expression is upregulated on TILs and blockade of LAG3 can enhance anti-tumour T cell responses [16,17]. BTLA belongs to CD28 family and is structurally similar to CTLA-4 and PD-1 [10]. BTLA expression on lymphocytes has been shown to attenuate T cell activation and proliferation. T cells from BTLA-deficient mice display a proliferative phenotype in response to T or B cell activation [18]. Expression of the negative regulator, programmed death ligand 1 (PD-L1) on tumor cells inhibits the activation of T cells upon binding to its receptor PD-1, thereby preventing effective anti-tumor immunity [19–22]. Monoclonal antibodies against PD-L1 have been explored in patients with cancer [21]. A previous study in our laboratory has shown that blockade of PD-L1 signaling enhanced anti-tumor effects in a melanoma tumor model [23]. Blockade of negative regulators on T cells in the tumor microenvironment may improve anti-tumor T cell responses and lead to improved immunotherapeutic strategies for cancer.

TIL therapy depends on the expansion of tumor-specific T cells from tumor fragments. Strategies to increase the number of T cells, expand reactive T cells at tumor site may improve and increase the probability of expanding tumor-specific T cells. In this study, we examined whether co-inhibitory blockade improves T cells for adoptive transfer and improves anti-tumor immune responses.

## Materials and Methods

### Animals

This study was carried out in strict accordance with the recommendations in the Guide for the Care and Use of Laboratory Animals of the National Institutes of Health. The protocol was reviewed and approved by the Institutional Animal Care and Use Committee at the University of South Florida (#A4100-01). Mice were humanely euthanized by CO_2_ inhalation according to the American Veterinary Medical Association Guidelines. Mice were observed daily and humanely euthanized if a solitary subcutaneous tumor exceeded 1.5cm in diameter or mice showed signs referable to metastatic cancer. All efforts were made to minimize suffering. Female C57BL.6 mice (6–8 weeks old) were purchased from Harlan Laboratories (Indianapolis, IN). Mice were housed at the Animal Research Facility of the H. Lee Moffitt Cancer Center and Research Institute.

### Tumor Cell Lines

B16 and MC38 murine colon cancer cell lines were maintained by serial *in vitro* passages in Complete medium (CM). CM consisted of RPMI 1640 supplemented with 10% heat-inactivated FCS, 0.1 mM nonessential amino acids, 1 mM sodium pyruvate, 2 mM fresh L-glutamine, 100 mg/ml streptomycin, 100 U/mL penicillin, 50 mg/mL gentamycin, 0.5 mg/mL fungizone (all from Life Technologies, Rockville, MD), and 0.05 mM 2-ME (Sigma-Aldrich, St. Louis, MO).

### Monoclonal Antibodies

The monoclonal antibodies anti-LAG3 (clone C9B7W), anti-BTLA (clone 6A6), anti-CTLA-4 (clone 9H10), anti-PD-1 (clone RMP1-14), and anti PDL-1 (clone 10F.9G2) were all purchased from BioXcell (West Lebanon, NH). Normal ratIgG or hamster IgG was used as isotype controls.

### *In Vivo* Treatment Model

A total of 1x10^5^ MC-38 colon cancer tumor cells were injected subcutaneously (s.c.) in C57BL/6 mice. Three days later, mice were injected with 300 ug of monoclonal antibodies (isotype, anti-PD-1, anti-PD-L1, anti-Lag-3, anti-CTLA-4 or anti-BTLA) intraperitoneally (i.p.). Mice continued to receive this treatment every 3–4 days until the tumor reached a size of 400mm^2^. Tumor size was measured and recorded every two days. In another set of experiments, mice were euthanatized at day 21 after tumor injection. Tumors and splenocytes were harvested for *in vitro* assays.

### Mouse TIL Isolation

Tumor cell suspensions were prepared from solid tumors by enzymatic digestion in HBSS (Life Technologies) containing 1 mg/ml collagenase, 0.1 mg/ml DNAse I, and 2.5 U/ml of hyaluronidase (all from Sigma-Aldrich) with constant stirring for 2 hours at room temperature. The resulting suspension was passed through a 70-um cell strainer, washed once with HBSS and resuspended in PBS + 3% BSA to a concentration of 1 x 10^6^ cells/ml for flow cytometric analysis. Cells were labeled with anti-CD90 microbeads according to the manufacturers’ instructions (Miltenyi Biotec) and purified using an autoMACS. After autoMACS purification, TIL were cultured for 5 days in the presence of IL-2 (3000 IU/ml). On day 5, TIL were collected for *in vitro* assays.

### Chromium Release Assay

A ^51^Cr release assay was done as described previously [3]. MC-38 cells were used as targets and B16 tumor cells were used as a control. TIL purified from tumors were used as effector cells. T cell purity was measured by flow cytometry and cells were 95% positive for CD90 (data not shown). Briefly, target MC-38 or B16 tumor cells were labeled with 100 μCi of ^51^Cr (Amersham Corp.) in 0.2 mL of medium at 37°C in a 5% CO_2_ atmosphere for one and a half hours. The labeled tumor cells were washed three times and added to the effector cells in triplicate wells of 96-well round-bottomed microplates at 50:1 and 25:1 effector to target ratios. After 5 hours, the percentage of specific ^51^Cr release was determined by the following equation: (experimental cpm − spontaneous cpm)/ (total cpm incorporated − spontaneous cpm) × 100. All determinations were done in triplicate, and the SE of all assays was calculated and was typically 5% of the mean or less.

### Elispot Assay

Briefly, TIL were plated at 1x10^4^ and co-cultured with 1x 10^3^ irradiated MC-38 or B16 tumor cells and incubated for 48 hours at 37°C. The number of IFN-γproducing cells in response to stimulation was evaluated in an ELISPOT assay. The number of spots was counted in triplicates and calculated using an automatic ELISPOT counter.

### Flow Cytometry

Spleens or TIL were harvested under sterile conditions. Single-cell suspensions were prepared, and red blood cells were removed using ACK lysis buffer. For analysis of immune cell populations, one million cells (splenocytes or tumor digest suspension) were incubated for 20 minutes on ice in staining medium with relevant antibodies for surface expression analysis according to the manufacturer’s instructions (all from BD Biosciences). Samples were analyzed using an LSRII (BD Biosciences) and the data was analyzed using FlowJo software (Tree Star).

### Adoptive Transfer of TIL

TIL were purified from either NrIgG or anti- PD-L1 antibody treated mice as described above. After autoMACS purification, TIL were cultured for 5 days in the presence of IL-2 (3000IU/ml) and TIL were used for *in vivo* studies. On day 4 following tumor injections, 5x10^6^ TIL per mouse were i.v. transferred. Beginning on day 4 and continuing every 12 hours for three days, mice also received 2.5e5 IU of IL-2 i.p. Following this treatment, tumor size was measured and recorded every 2 days.

### Statistical Analysis

A Mann-Whitney test (unpaired) or a Student’s t-test was used to compare between two treatment groups. All statistical evaluations of data were performed using Graph Pad Prism software. Statistical significance was achieved at p<0.05.

## Results

### TIL Isolated from Tumor Bearing Mice Are Tumor-Specific with High Cytotoxic Function

**Fig 1:**
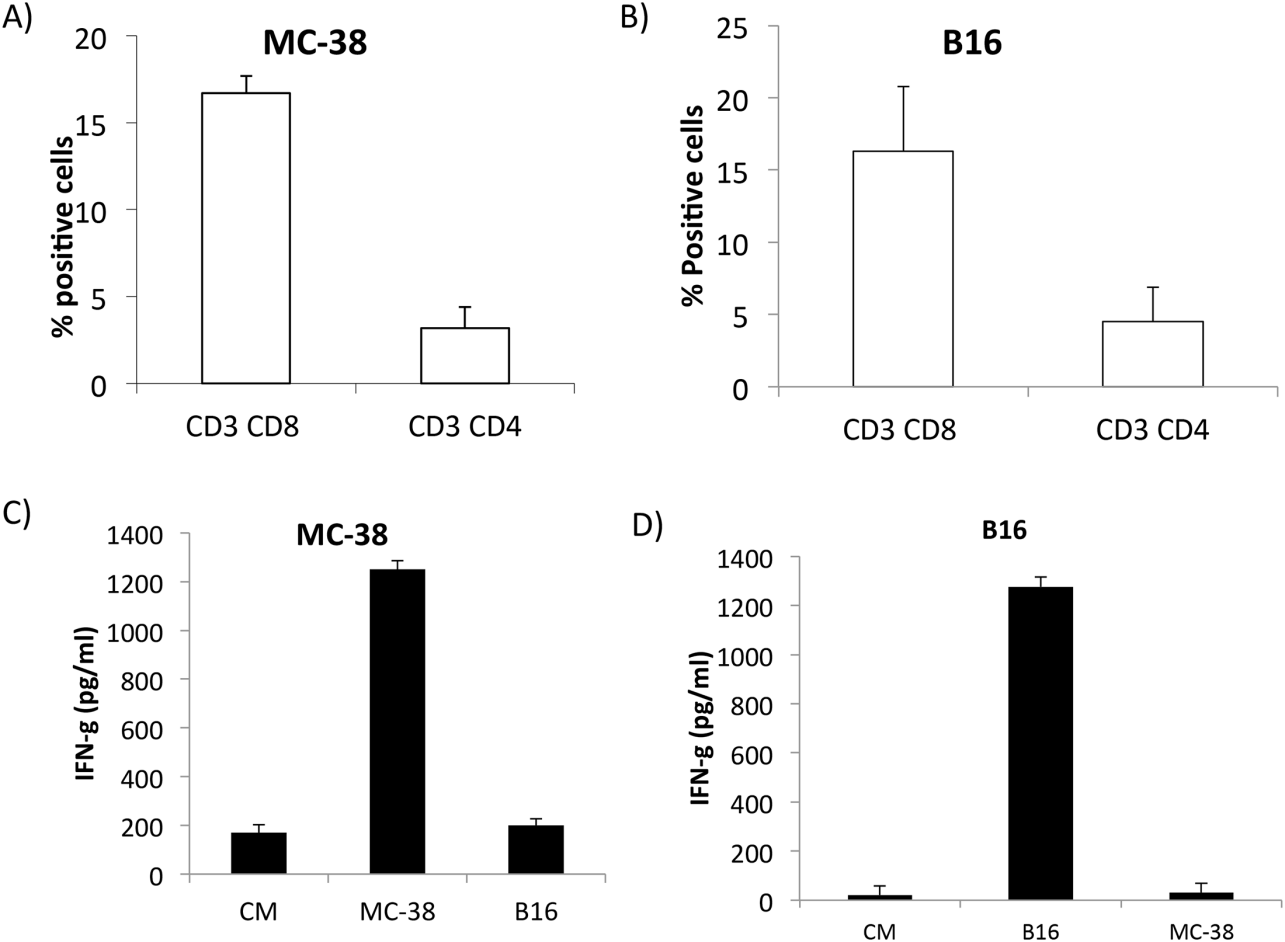
MC-38 and B16 TIL are tumor specific. A& B) Bar graph represents mean+ SD of CD8+ and CD4+ T cells infiltrating tumors (n = 8); C& D) MC-38 or B16 TIL were co-cultured with specific or irrelevant tumor cells for 48 hours. IFN-γ production was measured in culture supernatants by ELISA.

To examine the phenotype of infiltrating lymphocytes in tumors of B16 and MC-38-bearing mice, CD4+ and CD8+ T cells were analyzed by flow cytometry by gating on viable cells. As shown in Fig 1A and 1B, TIL isolated from MC-38 and B16 tumors had increased CD8+ T cell infiltration compared to CD4+ T cells. We next tested the tumor specificity and function of TIL isolated from MC-38 and B16 tumor bearing mice. TIL isolated from MC-38 (Fig 1C) and B16 (Fig 1D) when co-cultured in the presence of specific tumor cells had significant levels of IFN-γ production compared to irrelevant tumor cells. This data shows that TIL isolated from MC-38 and B16 tumors contain tumor-specific CD8+ T cells.

### Immune Checkpoint Receptors Expression on TIL

**Fig 2:**
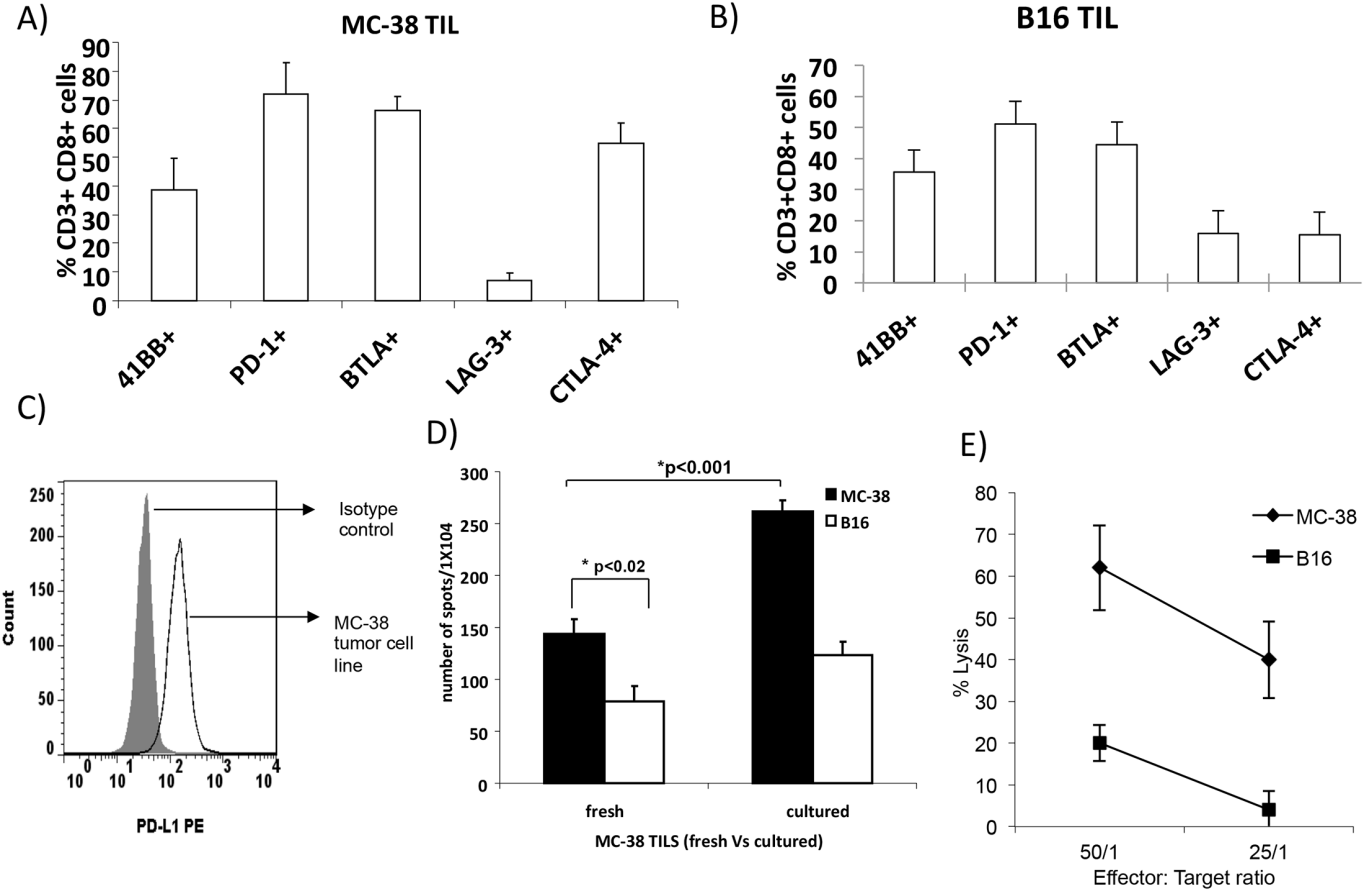
Expression of immune checkpoint receptors on MC-38 and B16 TIL. A&B) Flow cytometry analysis of PD-1, CTLA-4, BTLA, and LAG-3 expression on MC-38 (A) and B16 (B) TIL; C) PD-L1 expression on MC-38 tumor cells; D) fresh or cultured MC-38 TIL were co-cultured with MC-38 or irrelevant B16 tumor cells and incubated for 48 hours at 37°C. The number of IFN-γ producing cells in response to stimulation was evaluated in an ELISPOT assay. The number of spots was counted in triplicates and calculated using an automatic ELISPOT counter; E) TIL was cultured with ^51^Cr labeled MC-38 or B16 tumor cells at 50:1 and 25:1 effector to target ratios. After 5 hours, the percentage of specific ^51^Cr release was determined by the following equation: (experimental cpm − spontaneous cpm)/ (total cpm incorporated − spontaneous cpm) × 100. All determinations were done in triplicate, and the SE of all assays was calculated and was typically 5% of the mean or less.

Inhibitory receptors such as PD-1, CTLA-4, Lag-3 and BTLA and expressed on T cells and ligands such as PD-L1 have been shown to contribute to immune mediated suppression. We examined the expression of inhibitory immune checkpoint receptors on CD8+ T cells isolated from B16 and MC-38 tumors and measured expression of PD-1, CTLA-4, BTLA, and LAG-3. We also measured 41BB expression on TIL to determine activation status (Fig 2A and 2B). Reports have shown that PD-L1 expression on tumor cells mediates negative signaling through PD-1 interaction on T cells. Previous study from our lab has shown that B16 tumor cells express PD-L1 on their surface [23]. We examined PD-L1 expression on MC-38 tumor cells. As shown in Fig 2C, MC-38 expresses PD-L1.

### TIL Expansion *In Vitro*

We next examined the feasibility of expanding TIL *in vitro* from B16 and MC-38 tumors. C57/BL6 mice were injected with B16 or MC-38 and tumors were collected on day 21. Tumor cell suspensions were prepared from tumors by enzymatic digestion as described in methods section. Cells were labeled with anti-CD90 microbeads and purified using an autoMACS. After autoMACS purification, TIL were cultured for 5 days in the presence of IL-2 (3000 IU/ml). T cell purity was checked by flow cytometry and > 95% cells were positive for CD3. On day 5, TIL were collected, counted and used for *in vitro* assays TIL from MC-38 tumor expanded two-fold after 5 days of culture in media containing IL-2 (data not shown). However, we were not able to expand B16 TIL in culture for 5 days. Hence we utilized MC-38 tumor model for our TIL studies. An ELISPOT assay was performed to determine the number of IFN-γ producing TIL in response to stimulation by the MC-38 or irrelevant tumor cells. We compared freshly isolated TIL and TIL that were cultured for 5 days in media containing 3000 IU/ml IL-2. As shown in Fig 2D, both fresh and cultured TIL secreted IFN-γ in response to MC-38 cells. Significantly lower secretion of IFN-γ was measured in response to irrelevant B16 cells. Higher production of IFN-γ was measured in TIL cultured in the presence of IL-2 compared to fresh TIL (p<0.05). Next, we evaluated the ability of expanded TIL to mediate specific cell lysis *in vitro*. TIL isolated from MC-38 tumor bearing mice were cultured *in vitro* for 5 days in the presence of IL-2. On day 5, TIL were co-cultured with ^51^Cr-labeled MC-38 or B16 cells at effector to target ratios of 50:1 and 25:1. TIL exhibited cytotoxicity against MC-38 tumor cells at a 50:1 and 25:1 ratio and were tumor-specific (Fig 2E, p<0.001 compared to killing of irrelevant B16 cells). This data shows that cultured TIL mediates specific cytotoxicity against MC-38 cells.

### Inhibitory Immune Checkpoint Blockade Delays Tumor Growth in MC-38 Tumor Bearing Mice

**Fig 3:**
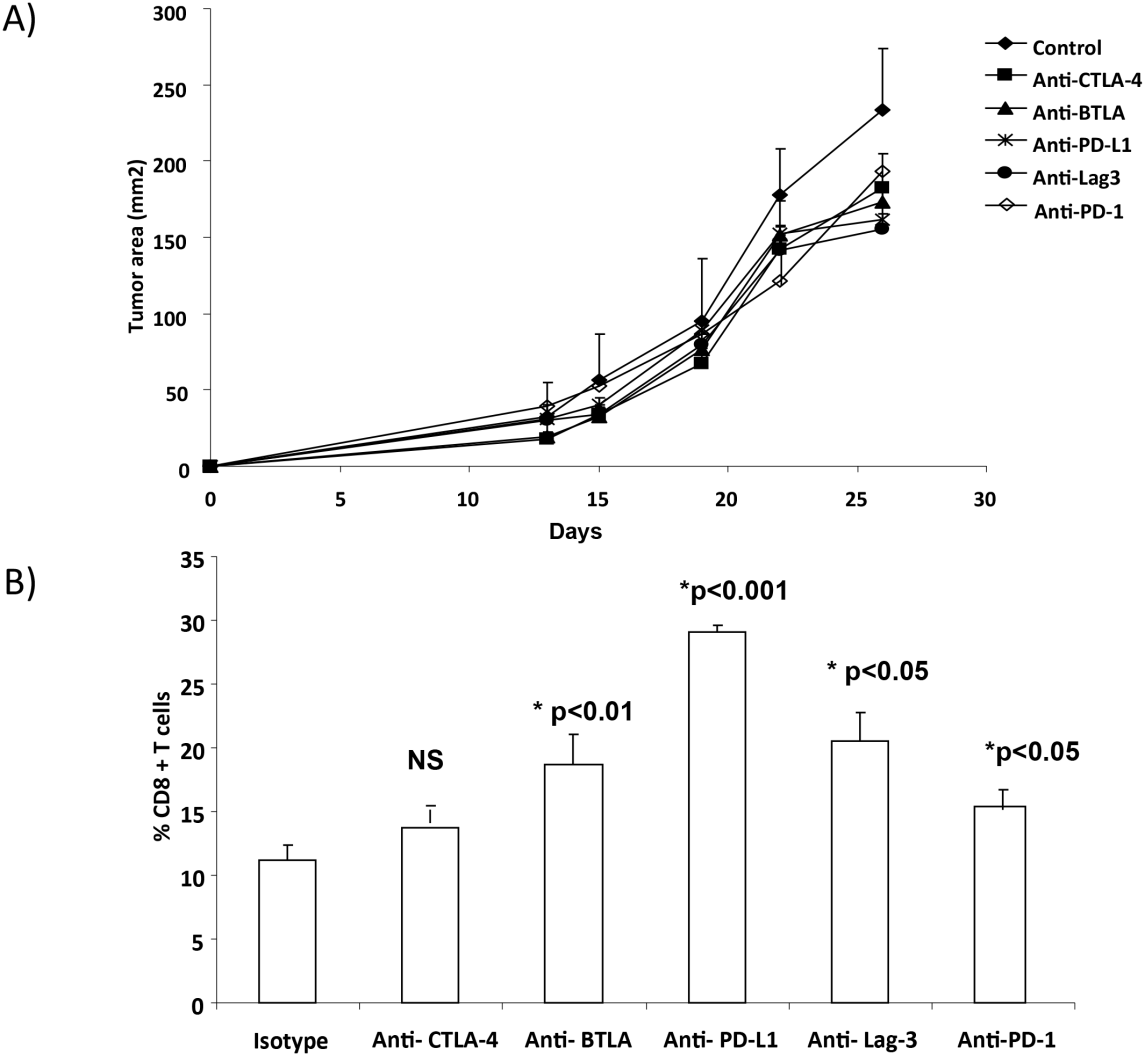
Inhibitory immune checkpoint blockade delays tumor growth in MC-38 tumor bearing mice. A total of 1x10^5^ MC-38 colon cancer tumor cells were injected subcutaneously (s.c.) in C57BL/6 mice. Three days later, mice were injected with 300 ug of monoclonal antibodies (isotype, anti-PD-1, anti-PD-L1, anti-Lag-3, anti-CTLA-4 or anti-BTLA) intraperitoneally (i.p.). Mice continued to receive this treatment every 3–4 days until the tumor reached a size of 400mm^2^. Tumor size was measured and recorded every two days. In another set of experiments, mice were euthanatized at day 21 after tumor injection. Tumors were harvested for *in vitro* assays A) Tumor growth B) Flow cytometry analysis of T cells infiltrating tumors after checkpoint blockade.

Based on the expression levels of inhibitory immune checkpoints, we examined whether blockade of these inhibitory immune checkpoints had an effect on TIL infiltration and tumor growth. As shown in Fig 3A, antibody treatment with anti-CTLA-4 (p<0.02), anti-BTLA (p<0.01), anti- PD-L1 (p<0.01), anti- Lag-3 (p<0.001) and anti- PD-1 antibodies (p<0.02) led to a modest but a significant delay in tumor growth in MC-38 bearing mice (p values compared to mice that received isotype control antibody). We next investigated whether blockade of individual co-inhibitory molecules had an effect on TIL infiltration that could contribute to the observed delay in MC-38 tumor growth. Tumors were collected on day 21 and CD8+ T cells within the TIL population were measured by flow cytometry. Treatment with anti-BTLA or anti-PD-L1, anti-Lag3 and anti-PD-1 led to a significant increase in CD8+ T cell infiltration compared to mice treated with isotype control antibody (Fig 3B). Tumor bearing mice that received anti-PD-L1 antibody demonstrated the most significant increase in T cell infiltration (p<0.001, compared isotype control). There was no significant increase in T cell infiltration in anti-CTLA-4 treated mice. Together, this data suggests that PD-L1 blockade is an effective strategy to increase CD8+ T cell infiltration in MC-38 tumors.

### PD-L1 Blockade Improves Tumor-Specific Immune Responses of TIL

**Fig 4:**
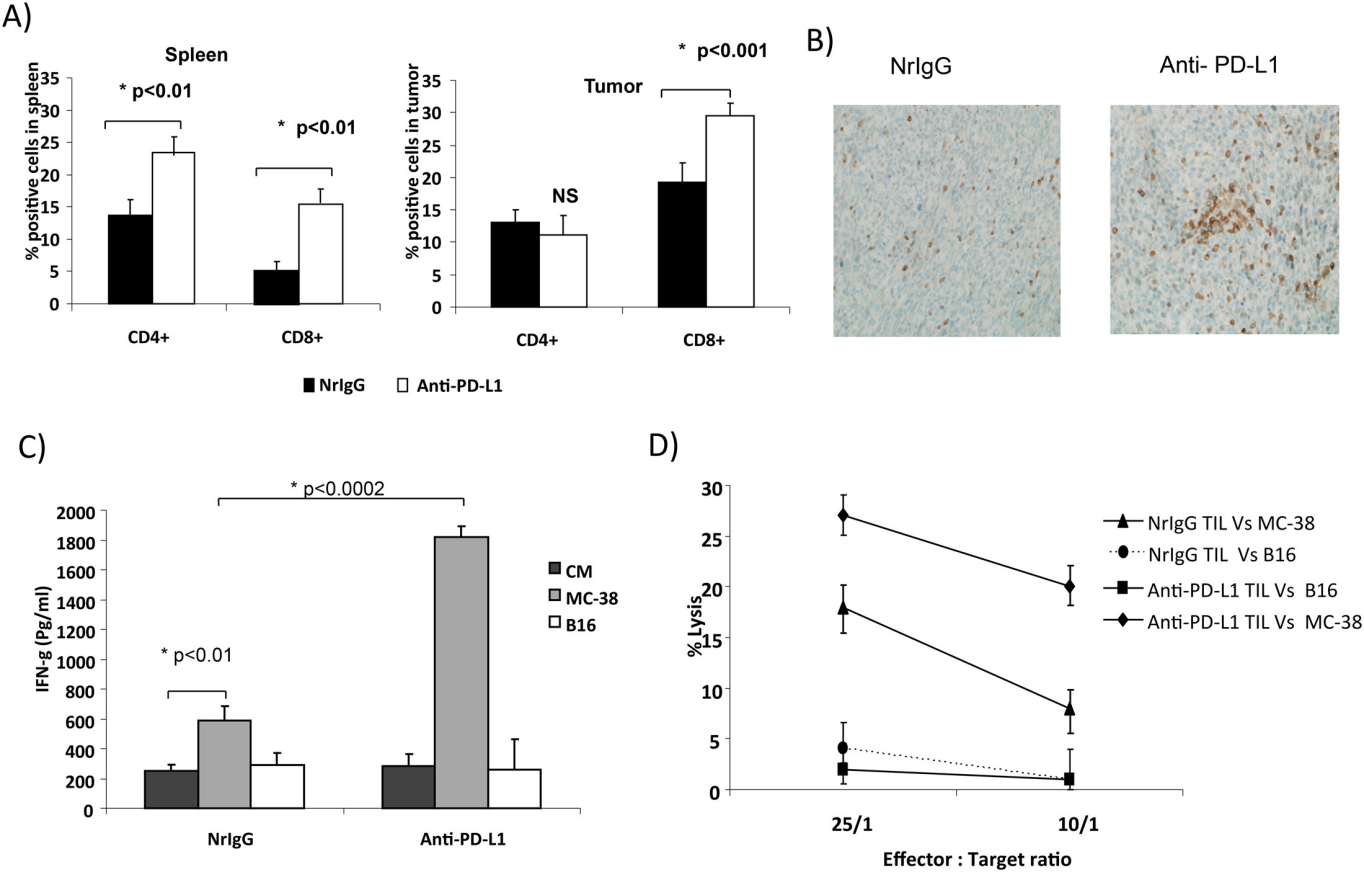
PD-L1 blockade increases T cell infiltration and enhances cytotoxic function of TIL. A) Bar graph represents CD4 and CD8+ T cell infiltration in spleen and tumors (n = 8); B) CD3+ T cell infiltration in tumor (n = 8); C) measurement of IFN-γ by ELISA; D) Percent of ^51^Cr release by TIL.

Since PD-L1 blockade led to a significant increase in T cell infiltration, we next examined whether PD-L1 blockade enhanced tumor-specific immune responses in MC-38 tumor bearing mice. Administration of anti-PD-L1 antibody enhanced the percentage of CD8+ T cells in the spleen (15.3%) and tumor (28.5%) compared to 5.6% of CD8+ T cells in the spleen and 17.6% of CD8+ T cells in tumor of mice treated with NrIgG (Fig 4A, n = 8). Similarly, anti-PD-L1 antibody treated mice had increased levels of CD4+ T cells in the spleen compared to NrIgG treated mice. No significant difference was measured in CD4+ T cell infiltration into tumors between mice treated with anti-PD-L1 antibody or NrIgG. Immunohistochemical staining revealed an increase in CD3+ T cell infiltration in the tumors of PD-L1 treated mice (Fig 4B, n = 8).

We next measured IFN-γ production in response to restimulation with tumor cells as a measure of T cell function. TIL isolated from tumors of NrIgG or anti-PD-L1 antibody treated mice were restimulated with MC-38 or irrelevant B16 cells for 48 hours. As shown in Fig 4C, TIL from anti-PD-L1 antibody treated tumor bearing mice demonstrated increased IFN-γ production in response to MC-38 cells (p<0.0002) compared to the TIL from NrIgG treated mice. This data suggests that PD-L1 blockade enhances TIL function in MC-38 tumor-bearing mice.

We next examined whether PD-L1 blockade improves the cytotoxic function of TIL. TIL isolated from tumors of anti-PD-L1 antibody or NrIgG treated mice were cultured in the presence of 3000IU/ml of IL-2. On day 5, TIL were analyzed for their cytotoxic function using a ^51^Cr release assay. Purified TIL from anti-PD-L1 antibody treated mice co-cultured with ^51^Cr-labeled MC-38 tumor cells had higher cytotoxicity (27%) at 25:1 and 10:1 (22%) ratios compared to the TIL from tumors of NrIgG treated mice (18% at 25:1 and 8%. at 10:1 ratio) (Fig 4D, p<0.01). No killing of irrelevant B16 cells was measured in either group. This data supports PD-L1 blockade to improve the cytotoxic function of TIL.

### PD-L1 Blockade Improves Anti-Tumor Efficacy of TIL *In Vivo*

**Fig 5:**
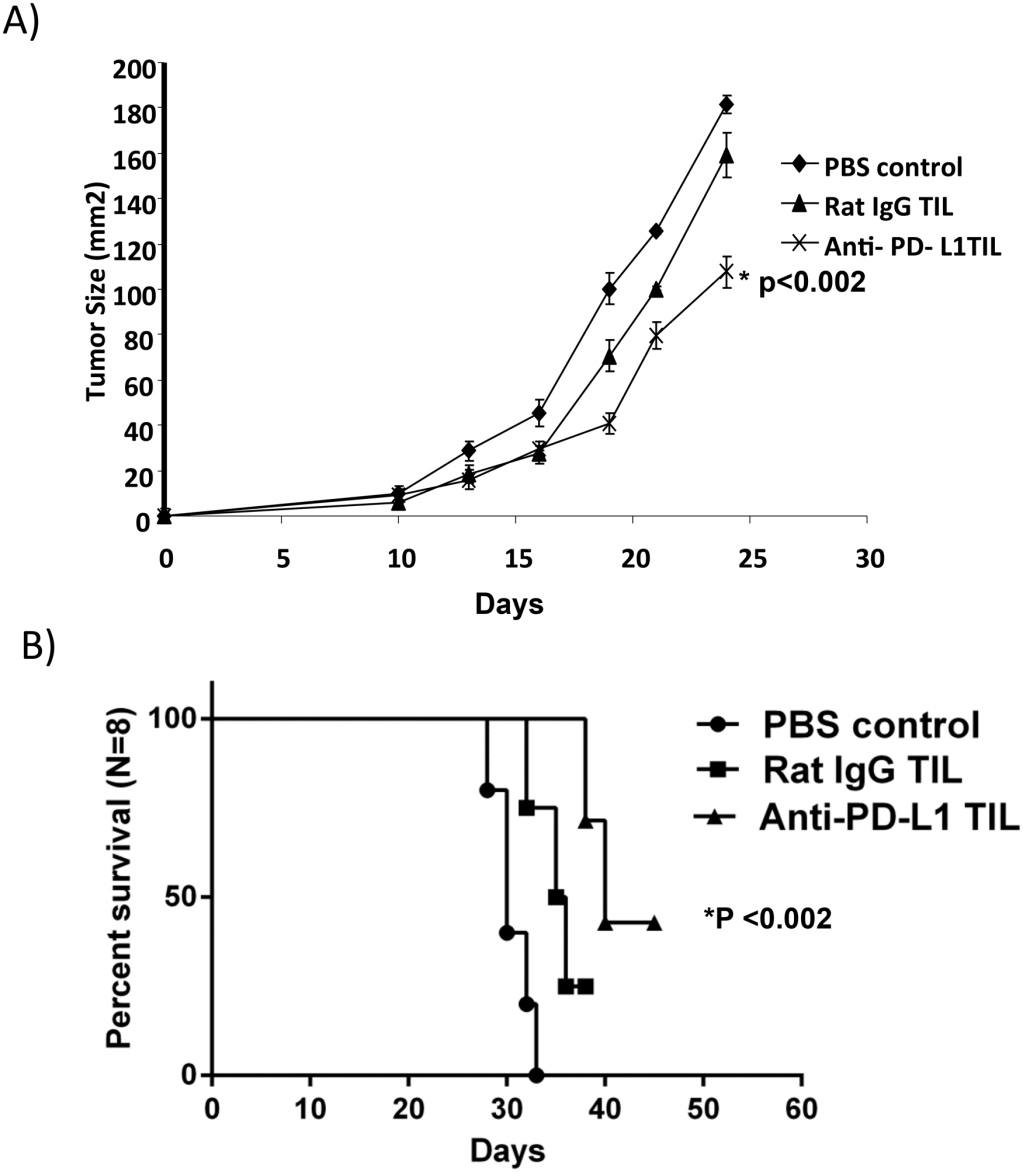
PD-L1 blockade improves the anti-tumor efficacy of TIL *in vivo*. A) Mice received MC-38 tumor cells on day 0 followed by 600 rad TBI on day 3. TIL isolated from NrIgG or anti-PD-L1 antibody treated mice (n = 8) were cultured with 3000IU/ml of IL-2 for 5 days and adoptively transferred on day 4 followed by 6 injections of 2.5e5 IU of IL-2 every 12 hours; B) survival curve (n = 8).

Next, we investigated PD-L1 blockade for the generation of TIL for adoptive cell therapy. Mice were injected s.c. with MC-38 tumor cells on day 0 followed by total body irradiation (TBI) with 600 rad on day 3. Cultured TIL from anti-PD-L1 or NrIgG antibody treated mice were adoptively transferred on day 4. As shown in Fig 5A, mice that received TIL derived from either NrIgG or anti-PD-L1 treated mice demonstrated a significant delay in tumor growth compared to the control mice that did not receive any TIL therapy (p<0.01). However, adoptive transfer of TIL from anti-PD-L1 antibody treated mice led to a significant delay in tumor growth and enhanced survival compared to mice that received TIL from NrIgG treated mice (Fig 5B, p<0.002). This data demonstrates that PD-L1 blockade improves TIL function that may enhance anti-tumor immune responses after adoptive transfer.

## Discussion

While TIL is a prognostic factor in cancer and is associated with prolonged survival rates in cancer patients, the infiltrating T cells are unable to induce total tumor regressions [2]. Adoptive transfer of infiltrating T cells has been a promising approach but is unable to induce total tumor regressions. This is due to the exhaustion of TIL that has been correlated with the expression of various immune checkpoint receptors on T cells and ligands by tumor cells and this may prevent TIL from inducing strong anti-tumor immune responses. Increased expression of co-inhibitory molecules can down regulate T cell activity and lead to tumor evasion. The purpose of this study is to restore the function of TIL for the use of adoptive cell therapy. In this study, blockade of Lag-3, BLTA, PD-1 or PD-L1 enhanced T cell infiltration into MC-38 tumors. However, the most significant increase in CD8+ T cell infiltration was seen following treatment with PD-L1 blocking antibodies. MC-38 tumor cells expressed high levels of PD-L1 and several reports support that PD-L1 expression on tumor cells can inhibit cytotoxic T lymphocytes through its interaction with the PD-1 receptor expressed by activated T cells [24,25]. The role of PD-1 and PD-L1 interactions in T cell exhaustion has been studied extensively. Expression of PD-1 on tumor-specific T cells leads to a profound impairment in the production of IL-2 that compromises their CTL function [26]. Blockade of the PD-1/PD-L1 interaction can reverse the T cell exhaustion and restore anti-tumor immunity [1,27]. A recent study has shown that anti-PD-L1 antibody treatment in combination with IL-15 reduces PD-1 expression on CD8^+^ T cells in murine metastatic colon cancer resulting in enhanced IFN-γ secretion and prolonged survival of tumor bearing animals [27]. Our laboratory has shown the therapeutic effect of PD-L1 blockade in improving DC vaccination and adoptive T cell transfer in the B16 melanoma model [23]. In this study, T cells isolated from MC-38 tumors after PD-L1 blockade produced IFN-γ in response to MC-38 cells and demonstrated increased CTL responses compared to the TIL from NrIgG-treated control mice. Our results show that blocking PD-L1 could restore the functional ability of TIL in inducing an anti-tumor immune response.

Although blockade of CTLA-4 delayed tumor growth in our model, we did not observe an increase in T cell infiltration into MC-38 tumor. CLTA-4 blockade has been shown to enhance anti-tumor immunity in tumor models when combined with other immunotherapies [28,29]. Dual blockade of PD-1 and CTLA-4 combined with a tumor vaccine restored T cell activity [30]. Hence, combination immunotherapies that include the blockade of CTLA-4 may improve T cell infiltration into tumors.

Among the immune checkpoint molecules measured on T cells, we observed low levels of Lag-3 expression on TIL compared to other co-inhibitory molecules. However, blockade of Lag-3 led to delayed growth of MC-38 tumors. While Lag-3 is expressed on CD4+ and CD8+ T cells, Lag-3 expression is well defined on an active CD4+CD25+Foxp3+ regulatory T cell subset. These cells have been shown to be highly suppressive and expanded in tumor sites [31]. A recent study has shown that pDCs also express high levels of Lag-3. Lag-3-mediated activation of pDCs within tumors may be in part responsible for directing an immune-suppressive environment [15]. It is possible that blockade of Lag-3 led to decreased suppression within the tumor that resulted in delayed tumor growth in the MC-38 model.

TIL-based therapies have been shown to induce effective anti-tumor immunity and tumor regression in various cancers [3,4,32,33]. TIL therapy depends on the expansion of tumor-specific T cells from tumor fragments. Strategies to improve reactive T cell infiltration and activation within tumors may increase the probability of expanding tumor-specific T cells for infusion. We show that blockade of PD-L1 led to improved infiltration and activation of anti-tumor T cells within tumors. Expansion *in vitro* and adoptive transfer of these T cells resulted in improved anti-tumor immunity. Although, we observed only a moderate delay in tumor growth in mice that received TIL from anti-PD-L1 treated mice, we believe that the treatment with anti-PD-L1 or other co-inhibitory antibodies after TIL transfer will enhance T cell persistence and efficacy. These studies are ongoing. Together, our results support co-inhibitory blockade prior to collection of tumor to generate TIL for adoptive cell therapy protocols for the treatment of cancer in clinical settings.

## Abbreviations

TIL: Tumor infiltrating lymphocytes

## References

[1] Wu X, Zhang H, Xing Q, Cui J, Li J, Li Y, et al PD-1(+) CD8(+) T cells are exhausted in tumours and functional in draining lymph nodes of colorectal cancer patients. Br J Cancer 111: 1391–1399. 10.1038/bjc.2014.416 25093496 PMC4183848

[2] Katz SC, Pillarisetty V, Bamboat ZM, Shia J, Hedvat C, Gonen M, et al (2009) T cell infiltrate predicts long-term survival following resection of colorectal cancer liver metastases. Ann Surg Oncol 16: 2524–2530. 10.1245/s10434-009-0585-3 19568816

[3] Pilon-Thomas S, Kuhn L, Ellwanger S, Janssen W, Royster E, Marzban S, et al Efficacy of adoptive cell transfer of tumor-infiltrating lymphocytes after lymphopenia induction for metastatic melanoma. J Immunother 35: 615–620. 22996367 10.1097/CJI.0b013e31826e8f5f PMC4467830

[4] Turcotte S, Gros A, Hogan K, Tran E, Hinrichs CS, Wunderlich JR, et al Phenotype and function of T cells infiltrating visceral metastases from gastrointestinal cancers and melanoma: implications for adoptive cell transfer therapy. J Immunol 191: 2217–2225. 10.4049/jimmunol.1300538 23904171 PMC3748336

[5] Dudley ME, Yang JC, Sherry R, Hughes MS, Royal R, Kammula U, et al (2008) Adoptive cell therapy for patients with metastatic melanoma: evaluation of intensive myeloablative chemoradiation preparative regimens. J Clin Oncol 26: 5233–5239. 10.1200/JCO.2008.16.5449 18809613 PMC2652090

[6] Dudley ME, Wunderlich JR, Shelton TE, Even J, Rosenberg SA (2003) Generation of tumor-infiltrating lymphocyte cultures for use in adoptive transfer therapy for melanoma patients. J Immunother 26: 332–342. 12843795 10.1097/00002371-200307000-00005 PMC2305721

[7] Curran MA, Kim M, Montalvo W, Al-Shamkhani A, Allison JP Combination CTLA-4 blockade and 4-1BB activation enhances tumor rejection by increasing T-cell infiltration, proliferation, and cytokine production. PLoS One 6: e19499 10.1371/journal.pone.0019499 21559358 PMC3085474

[8] Gros A, Robbins PF, Yao X, Li YF, Turcotte S, Tran E, et al PD-1 identifies the patient-specific CD8(+) tumor-reactive repertoire infiltrating human tumors. J Clin Invest 124: 2246–2259. 10.1172/JCI73639 24667641 PMC4001555

[9] Mocellin S, Nitti D CTLA-4 blockade and the renaissance of cancer immunotherapy. Biochim Biophys Acta 1836: 187–196. 10.1016/j.bbcan.2013.05.003 23748107

[10] Watanabe N, Gavrieli M, Sedy JR, Yang J, Fallarino F, Loftin SK, et al (2003) BTLA is a lymphocyte inhibitory receptor with similarities to CTLA-4 and PD-1. Nat Immunol 4: 670–679. 12796776 10.1038/ni944

[11] Woo SR, Turnis ME, Goldberg MV, Bankoti J, Selby M, Nirschl CJ, et al Immune inhibitory molecules LAG-3 and PD-1 synergistically regulate T-cell function to promote tumoral immune escape. Cancer Res 72: 917–927. 10.1158/0008-5472.CAN-11-1620 22186141 PMC3288154

[12] Weber J, Hamid O, Amin A, O'Day S, Masson E, Goldberg SM, et al Randomized phase I pharmacokinetic study of ipilimumab with or without one of two different chemotherapy regimens in patients with untreated advanced melanoma. Cancer Immun 13: 7 23833564 PMC3700777

[13] Triebel F (2003) LAG-3: a regulator of T-cell and DC responses and its use in therapeutic vaccination. Trends Immunol 24: 619–622. 14644131 10.1016/j.it.2003.10.001

[14] Huang CT, Workman CJ, Flies D, Pan X, Marson AL, Zhou G, et al (2004) Role of LAG-3 in regulatory T cells. Immunity 21: 503–513. 15485628 10.1016/j.immuni.2004.08.010

[15] Camisaschi C, De Filippo A, Beretta V, Vergani B, Villa A, Vergani E, et al Alternative activation of human plasmacytoid DCs in vitro and in melanoma lesions: involvement of LAG-3. J Invest Dermatol 134: 1893–1902. 10.1038/jid.2014.29 24441096

[16] Demeure CE, Wolfers J, Martin-Garcia N, Gaulard P, Triebel F (2001) T Lymphocytes infiltrating various tumour types express the MHC class II ligand lymphocyte activation gene-3 (LAG-3): role of LAG-3/MHC class II interactions in cell-cell contacts. Eur J Cancer 37: 1709–1718. 11527700 10.1016/s0959-8049(01)00184-8

[17] Grosso JF, Kelleher CC, Harris TJ, Maris CH, Hipkiss EL, De Marzo A, et al (2007) LAG-3 regulates CD8+ T cell accumulation and effector function in murine self- and tumor-tolerance systems. J Clin Invest 117: 3383–3392. 17932562 10.1172/JCI31184 PMC2000807

[18] Serriari NE, Gondois-Rey F, Guillaume Y, Remmerswaal EB, Pastor S, Messal N, et al (2010) B and T lymphocyte attenuator is highly expressed on CMV-specific T cells during infection and regulates their function. J Immunol 185: 3140–3148. 10.4049/jimmunol.0902487 20693422

[19] Topalian SL, Hodi FS, Brahmer JR, Gettinger SN, Smith DC, McDermott DF, et al Safety, activity, and immune correlates of anti-PD-1 antibody in cancer. N Engl J Med 366: 2443–2454. 10.1056/NEJMoa1200690 22658127 PMC3544539

[20] Lipson EJ, Sharfman WH, Drake CG, Wollner I, Taube JM, Anders RA, et al Durable cancer regression off-treatment and effective reinduction therapy with an anti-PD-1 antibody. Clin Cancer Res 19: 462–468. 10.1158/1078-0432.CCR-12-2625 23169436 PMC3548952

[21] Brahmer JR, Tykodi SS, Chow LQ, Hwu WJ, Topalian SL, Hwu P, et al Safety and activity of anti-PD-L1 antibody in patients with advanced cancer. N Engl J Med 366: 2455–2465. 10.1056/NEJMoa1200694 22658128 PMC3563263

[22] Brahmer JR, Drake CG, Wollner I, Powderly JD, Picus J, Sharfman WH, et al Phase I study of single-agent anti-programmed death-1 (MDX-1106) in refractory solid tumors: safety, clinical activity, pharmacodynamics, and immunologic correlates. J Clin Oncol 28: 3167–3175. 10.1200/JCO.2009.26.7609 20516446 PMC4834717

[23] Pilon-Thomas S, Mackay A, Vohra N, Mule JJ Blockade of programmed death ligand 1 enhances the therapeutic efficacy of combination immunotherapy against melanoma. J Immunol 184: 3442–3449. 10.4049/jimmunol.0904114 20194714 PMC2913584

[24] Maier H, Isogawa M, Freeman GJ, Chisari FV (2007) PD-1:PD-L1 interactions contribute to the functional suppression of virus-specific CD8+ T lymphocytes in the liver. J Immunol 178: 2714–2720. 17312113 10.4049/jimmunol.178.5.2714

[25] Abiko K, Mandai M, Hamanishi J, Yoshioka Y, Matsumura N, Baba T, et al PD-L1 on tumor cells is induced in ascites and promotes peritoneal dissemination of ovarian cancer through CTL dysfunction. Clin Cancer Res 19: 1363–1374. 10.1158/1078-0432.CCR-12-2199 23340297

[26] Sakuishi K, Apetoh L, Sullivan JM, Blazar BR, Kuchroo VK, Anderson AC Targeting Tim-3 and PD-1 pathways to reverse T cell exhaustion and restore anti-tumor immunity. J Exp Med 207: 2187–2194. 10.1084/jem.20100643 20819927 PMC2947065

[27] Yu P, Steel JC, Zhang M, Morris JC, Waldmann TA Simultaneous blockade of multiple immune system inhibitory checkpoints enhances antitumor activity mediated by interleukin-15 in a murine metastatic colon carcinoma model. Clin Cancer Res 16: 6019–6028. 10.1158/1078-0432.CCR-10-1966 20924130 PMC3005104

[28] Kocak E, Lute K, Chang X, May KF Jr., Exten KR, Zhang H, et al (2006) Combination therapy with anti-CTL antigen-4 and anti-4-1BB antibodies enhances cancer immunity and reduces autoimmunity. Cancer Res 66: 7276–7284. 16849577 10.1158/0008-5472.CAN-05-2128

[29] Son CH, Bae JH, Shin DY, Lee HR, Choi YJ, Jo WS, et al CTLA-4 blockade enhances antitumor immunity of intratumoral injection of immature dendritic cells into irradiated tumor in a mouse colon cancer model. J Immunother 37: 1–7. 24316550 10.1097/CJI.0000000000000007

[30] Duraiswamy J, Kaluza KM, Freeman GJ, Coukos G Dual blockade of PD-1 and CTLA-4 combined with tumor vaccine effectively restores T-cell rejection function in tumors. Cancer Res 73: 3591–3603. 10.1158/0008-5472.CAN-12-4100 23633484 PMC3686913

[31] Camisaschi C, Casati C, Rini F, Perego M, De Filippo A, Triebel F, et al LAG-3 expression defines a subset of CD4(+)CD25(high)Foxp3(+) regulatory T cells that are expanded at tumor sites. J Immunol 184: 6545–6551. 10.4049/jimmunol.0903879 20421648

[32] Chacon JA, Pilon-Thomas S, Sarnaik AA, Radvanyi LG Continuous 4-1BB co-stimulatory signals for the optimal expansion of tumor-infiltrating lymphocytes for adoptive T-cell therapy. Oncoimmunology 2: e25581 24319633 10.4161/onci.25581 PMC3850170

[33] Turcotte S, Gros A, Tran E, Lee CC, Wunderlich JR, Robbins PF, et al Tumor-reactive CD8+ T cells in metastatic gastrointestinal cancer refractory to chemotherapy. Clin Cancer Res 20: 331–343. 10.1158/1078-0432.CCR-13-1736 24218514 PMC3927404

